# Inhibition Profiling of *Rhodiola crenulata* on Lactate Dehydrogenase Isoenzymes

**DOI:** 10.3390/molecules30214199

**Published:** 2025-10-27

**Authors:** Yi-Xin Wei, Motahareh Asgari, Qun-Fei Zhao, Shu-Sheng Zhang, Zuliayi Alifujiang, Fang Zhang, Xiu-Ping Chen, Chen-Guo Feng

**Affiliations:** The Research Center of Chiral Drugs, Shanghai Frontiers Science Center for TCM Chemical Biology, Innovation Research Institute of Traditional Chinese Medicine, Shanghai University of Traditional Chinese Medicine, 1200 Cailun Road, Shanghai 201203, China

**Keywords:** *Rhodiola crenulata*, lactate dehydrogenase isoenzymes, inhibition profiling, differential inhibition

## Abstract

Although *Rhodiola crenulata* (*R. crenulata*) exhibits anti-tumor effects, its mechanism of action has yet to be elucidated. Lactate dehydrogenase (LDH), a key glycolytic enzyme in tumor metabolism, has emerged as a promising target for anticancer drug development. To elucidate the anticancer mechanism of *R. crenulata*, this study systematically screened its bioactive components for inhibitory activity against LDHA and LDHB subunits. First, the main components of *R. crenulata* were identified using HPLC-QTOF MS. Then, the inhibitory potency of these identified components was assessed against recombinant LDHA and LDHB. Finally, isoenzyme inhibition of the bioactive components was elucidated through structure-based molecular docking and cell viability assays. The results showed that five *R. crenulata* compounds—quercetin, luteolin, kaempferol, epicatechin gallate, and ellagic acid—showed significant LDH inhibition, with stronger effects on LDHA than on LDHB. Against LDHA, the IC_50_ values for quercetin, luteolin, kaempferol, and epicatechin gallate were 0.27 ± 0.02 µM, 1.19 ± 0.09 µM, 0.70 ± 0.13 µM, and 2.27 ± 0.23 µM, respectively. Against LDHB, the values for quercetin, luteolin, and kaempferol were 0.87 ± 0.07 µM, 2.71 ± 0.39 µM, and 8.69 ± 0.85 µM, respectively. Molecular docking simulations and cell viability assays of the five bioactive compounds revealed their interactions with LDH subunits and supported their inhibitory effects. This study provides the first comprehensive inhibition profile of *R. crenulata* targeting LDH isoenzymes. It underscores the potential of *R. crenulata* in LDH-targeted therapeutics and supports its further development for cancer treatment.

## 1. Introduction

Cancer has garnered significant and growing attention as a high-priority research area within global scientific and medical communities. A defining hallmark of cancer is metabolic reprogramming, wherein cells rewire nutrient utilization to sustain rapid proliferation and survival [1]. This reprogramming encompasses heightened glutaminolysis, aberrant lipid synthesis, and, most prominently, a shift toward aerobic glycolysis—the Warburg effect [1,2]. By accelerating glycolytic flux, tumors generate intermediates for macromolecular synthesis and organelle biogenesis, thereby sustaining tumor expansion [3]. A key player in cancer metabolism is lactate dehydrogenase (LDH), the enzyme responsible for the final step of aerobic glycolysis, catalyzing the reversible interconversion of pyruvate and lactate while simultaneously mediating the redox cycling of NADH and NAD^+^ [4].

Structurally, LDH functions as a tetramer primarily composed of the LDHA (M subunit) and LDHB (H subunit) subunits, which assemble into five distinct isoenzymes: LDH-1 (H_4_), LDH-2 (M_1_H_3_), LDH-3 (M_2_H_2_), LDH-4 (M_3_H_1_), and LDH-5 (M_4_) [5]. While LDHA and LDHB share similar tertiary structures, variations in charged residues near the active sites confer them distinct kinetic properties [6]. LDHA exhibits higher catalytic efficiency for pyruvate reduction to lactate, while LDHB preferentially drives lactate oxidation back to pyruvate [5]. During catalysis, each subunit functions independently, simultaneously binding one substrate molecule and one coenzyme molecule to complete the redox reaction. Elevated LDHA expression has been widely detected in diverse malignancies, where it drives tumorigenic processes including uncontrolled proliferation, malignant maintenance, and invasion [7,8,9,10]. Unlike the consistently tumorigenic role of LDHA, LDHB displays significant functional plasticity across cancers [7,11]. For example, LDHB exhibits tumor-promoting effects in pancreatic cancer [12,13], breast cancer [14,15], lung cancer [16], and colorectal cancer [17,18], while demonstrating tumor-suppressive activity in prostate cancer [19,20] and liver cancer [21,22].

Given the crucial role of LDH in cancer metabolism, there has been increasing interest in developing LDH-targeted antitumor drugs. However, no clinically effective inhibitors have been developed to date, and most reported compounds exhibit poor subunit selectivity [23]. Despite the high structural conservation between LDHA and LDHB, their distinct biological functions and expression across tumor types suggest that designing subunit-selective inhibitors offers a promising strategy for developing clinically viable LDH inhibitors.

Herbal medicines are generally valued for their gentle bioactivity, low toxicity, and good clinical tolerability [24]. These distinctive properties have driven growing interest in target-based screening of bioactive constituents from herbal medicines. Nevertheless, the conventional approach of screening inhibitors extensively against the drug database fails to capture the holistic therapeutic principles and synergistic effects that characterize herbal medicine therapy. Currently, multiple technologies have been developed to address this challenge, including radioligand binding assays [25], affinity mass spectrometry [26], and computer-aided virtual screening [27]. A critical limitation persists, as these technologies primarily assess binding thermodynamics rather than functional bioactivity, resulting in substantial false-positive identification rates [28,29].

*R. crenulata* is a perennial herbaceous plant native to high-altitude regions. Several contemporary studies have validated its therapeutic applications in cardiovascular dysfunction [30,31], renal impairment [32], neurodegenerative disorders [33,34], hepatic fibrogenesis [35], immune dysregulation [36], and malignant tumors [37,38,39]. However, these studies have predominantly attributed the antitumor effects of *R. crenulata* to isolated constituents (e.g., salidroside), rather than systematically elucidating its complex polypharmacological mechanisms of action [40].

To systematically elucidate the anticancer mechanisms and bioactive constituents of *R. crenulata*, we selected LDH isoenzymes as targets for profiling its inhibitory activity. After characterizing the isoenzyme inhibition of the *R. crenulata* extract, we employed HPLC-QTOF MS to identify its main constituents and evaluated their individual enzymatic inhibitory effects using the enzyme activity assay. The inhibition of the screened bioactive components was further elucidated through structure-based molecular docking and cell viability assays.

## 2. Results and Discussion

### 2.1. Substrate-Specific Kinetic Parameters of LDHA and LDHB

**Figure 1:**
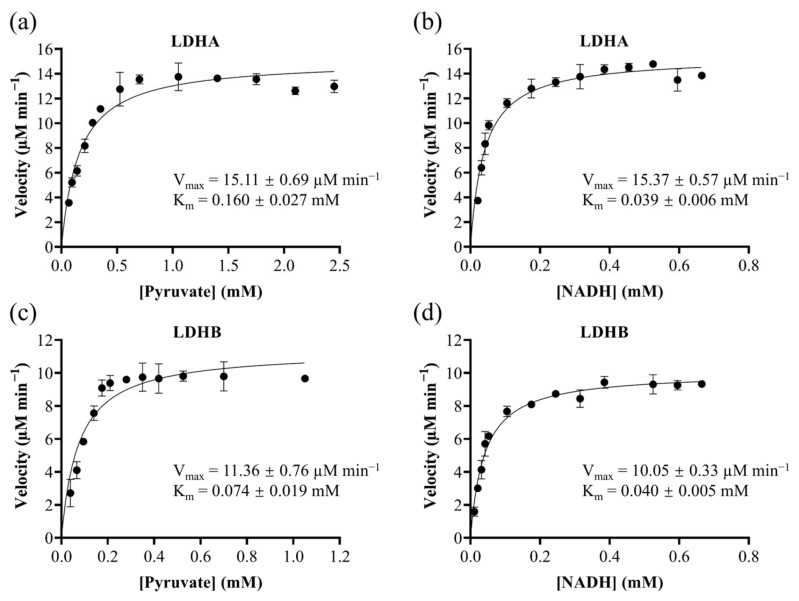
Michaelis-Menten kinetics of recombinant LDHA and LDHB. Kinetics of LDHA toward pyruvate (**a**) and NADH (**b**); kinetics of LDHB toward pyruvate (**c**) and NADH (**d**).

LDH catalyzes the reversible interconversion of pyruvate and lactate while mediating the redox cycling of NADH and NAD^+^. To characterize the enzymatic properties of recombinant LDHA and LDHB, we monitored their activities toward pyruvate and NADH by measuring absorbance changes at 340 nm. Initial reaction velocities were determined from progress curves generated across a range of substrate concentrations. Subsequent Michaelis-Menten analysis, performed by fitting the velocity versus substrate concentration plots (Figure 1), yielded the kinetic parameters (K_m_ and V_max_). Notably, the K_m_ value serves as an inverse indicator of substrate binding affinity, with lower values reflecting stronger enzyme-substrate interactions.

LDHA displayed K_m_ values of 0.160 ± 0.027 mM for pyruvate and 0.039 ± 0.006 mM for NADH, with corresponding V_max_ values of 15.11 ± 0.69 μM/min and 15.37 ± 0.57 μM/min. For LDHB, the K_m_ values were 0.074 ± 0.019 mM for pyruvate and 0.040 ± 0.005 mM for NADH, with V_max_ values of 11.36 ± 0.76 μM/min and 10.05 ± 0.33 µM/min, respectively. Although the K_m_ values for NADH were nearly equal between LDHA and LDHB, LDHA showed a significantly higher K_m_ for pyruvate compared to LDHB, indicating stronger binding affinity of LDHB for pyruvate. These results are consistent with LDHB’s physiological role in oxidizing lactate back to pyruvate [41,42]. The higher V_max_ values for LDHA indicate its specialization in rapid pyruvate reduction during glycolysis. Collectively, these findings demonstrate that while LDHA is optimized for glycolytic pyruvate reduction, LDHB is specialized for efficient pyruvate binding under aerobic conditions. Furthermore, these results suggest that inhibitors targeting the pyruvate-binding site are more likely to achieve isoenzyme-selective inhibition.

### 2.2. Validation of the LDHA and LDHB Inhibitor Screening Platforms

To ensure the reliable identification of inhibitors and minimize false positives, we rigorously validated the screening platform using established quality metrics. The Z′ factor, a critical parameter for assessing assay robustness, was used to evaluate our screening platform. The obtained values of 0.94 for LDHA and 0.82 for LDHB demonstrate excellent system performance, as they significantly exceed the accepted threshold of 0.5 for robust assays [43] and satisfy the more stringent criterion (Z′ > 0.7) for high-throughput screening. These high Z′ values indicated the high signal-to-background ratio and minimal well-to-well variability of the screening platform.

**Figure 2:**
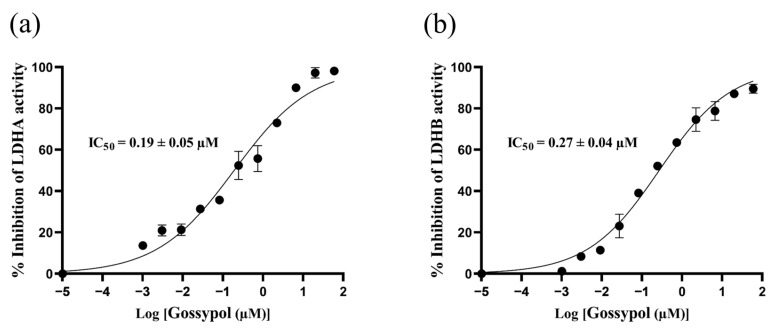
Dose-response curves of gossypol against LDHA (**a**) and LDHB (**b**).

For pharmacological validation, gossypol (a benchmark LDH inhibitor) was employed as a reference inhibitor. Dose-response curves were generated by plotting percentage inhibition against the logarithm of inhibitor concentrations (log_10_[gossypol]), with IC_50_ values determined by nonlinear regression analysis. Quantitative analysis yielded IC_50_ values of 0.19 ± 0.05 µM for LDHA and 0.27 ± 0.04 µM for LDHB (Figure 2). The submicromolar inhibitory potency observed for both isoforms demonstrates the platform’s sensitivity in detecting biologically relevant inhibition and confirms its utility for high-throughput screening applications.

### 2.3. Differential Inhibitory Effects of R. crenulata Extract on LDHA and LDHB

**Figure 3:**
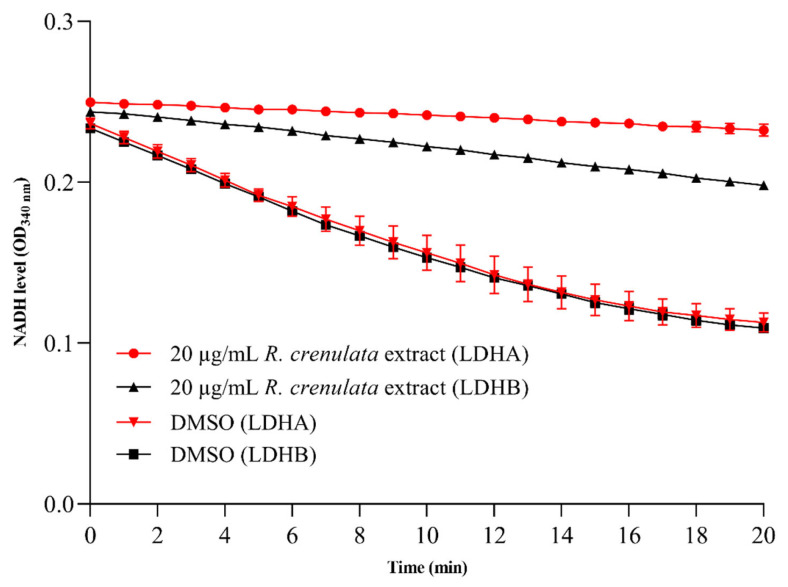
Enzymatic progress curves of *R. crenulata* extract against LDHA and LDHB. Progress curves of NADH consumption demonstrate significant, time-dependent inhibition of both isoforms (*p* < 0.0001) relative to the DMSO control.

While some isolated components (e.g., salidroside) have been extensively studied for antitumor effects, the antitumor potential of *R. crenulata* extract still remains insufficiently investigated. Given the crucial role of LDH in cancer metabolism, we evaluated the inhibitory effects of *R. crenulata* extract on both LDHA and LDHB. As shown in Figure 3, enzyme activity was monitored in real time by measuring the absorbance decrease at 340 nm, which reflects NADH consumption. Comparative analysis of enzymatic progress curves revealed that dimethyl sulfoxide (DMSO)-treated controls maintained high, stable activity throughout the entire recording window, whereas the extract induced a time-dependent decline in both isoforms. In addition, *R. crenulata* extract showed greater inhibition of LDHA compared to LDHB. Based on the inhibition rate formula, the extract exhibited differential inhibitory effects of 90.9 ± 2.6% on LDHA and 73.1 ± 0.8% on LDHB. These results indicate that some bioactive components in *R. crenulata* differentially target LDH subunits, with significantly greater efficacy against LDHA. Therefore, it is valuable to identify the specific inhibitory constituents in *R. crenulata* and evaluate their therapeutic potential.

### 2.4. Constituent Profiling of R. crenulata

**Figure 4:**
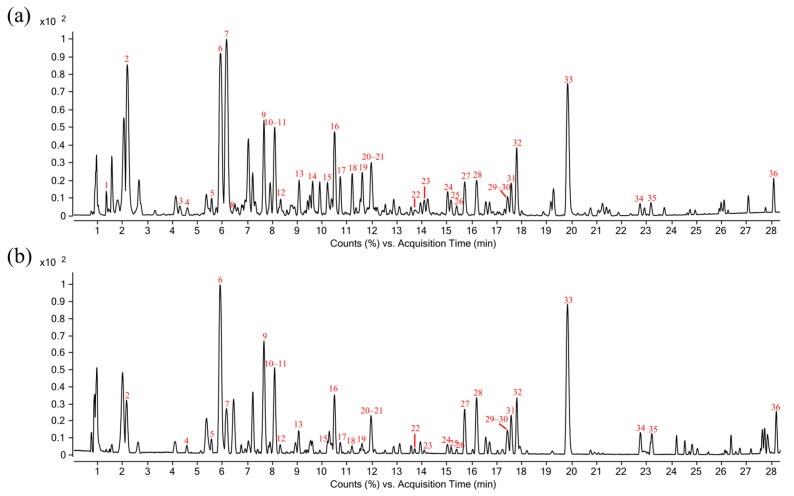
BPCs of *R. crenulata* extract in (**a**) negative and (**b**) positive ionization modes.

*R. crenulata* is known to biosynthesize a diverse array of bioactive metabolites, including phenylethanoid glycosides, flavonoids, organic acids, phenylpropanoids, and polyphenols [44,45]. To systematically characterize its chemical constituents, we performed an untargeted metabolomic analysis using HPLC-QTOF MS with data-dependent acquisition. The Base Peak Chromatograms (BPCs) acquired in both positive and negative ionization modes showed a complex metabolite profile in Figure 4. By comparing acquired mass spectra against chemical standards, mass spectral databases (e.g., GNPS), and published literature, we successfully identified and annotated 36 major bioactive compounds. The complete chemical profile of these compounds—including retention times (RT), precursor ions and their mass error, characteristic fragment ions (MS/MS), and molecular formulas—is comprehensively detailed in Table 1.

### 2.5. Identification of LDHA and LDHB Inhibitors from R. crenulata Extract

Given the differential expression patterns of LDHA and LDHB observed across different cancer types, these subunits emerge as promising candidates for tissue-specific targeted therapies. The structural complexity of LDH isoenzymes *in vivo*, which form five distinct tetrameric combinations, presents significant challenges for the development of selective inhibitors [53]. In this study, recombinant LDHA and LDHB were employed to effectively circumvent the heterogeneity of mixed tetramers found in physiological contexts. Based on these recombinant proteins, we established a screening platform and evaluated its robustness, sensitivity, and applicability. As individual subunits within the LDH tetramer retain independent catalytic activity, inhibitors targeting specific subunits identified by this platform represent a promising therapeutic strategy.

**Figure 5:**
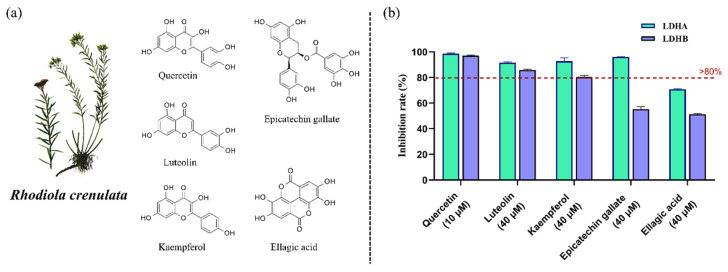
Structures and inhibitory activity of five bioactive components from *R. crenulate*. (**a**) Chemical structures of the five bioactive components. (**b**) Inhibitory effects of the five bioactive components against LDHA and LDHB.

**Figure 6:**
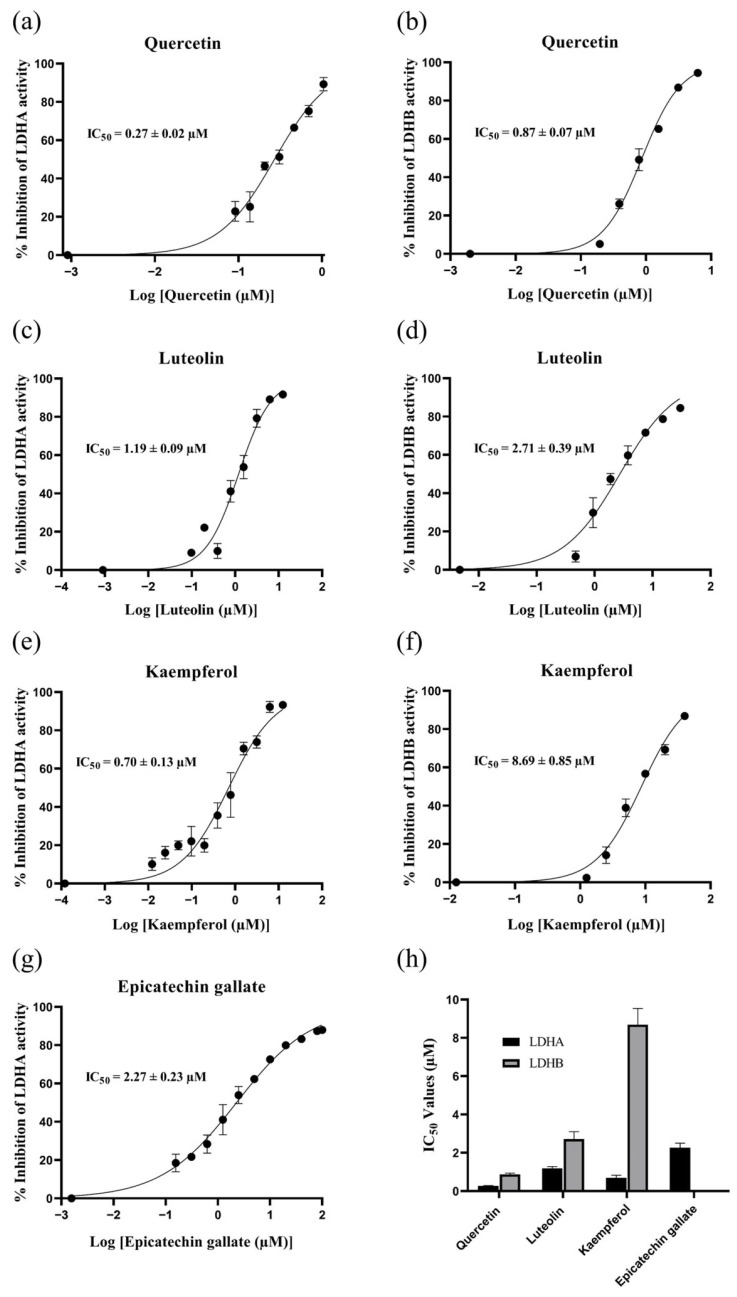
Dose-response curves for quercetin, luteolin, kaempferol, and epicatechin gallate against LDHA (**a**,**c**,**e**,**g**) and for quercetin, luteolin, and kaempferol against LDHB (**b**,**d**,**f**); (**h**) comparison of their IC_50_ values.

As preliminary screening revealed that the *R. crenulata* crude extract differentially inhibited LDHA and LDHB, we employed this validated platform to screen the extract for specific LDH inhibitors. Using this platform, we assessed 19 commercially available standards corresponding to identified metabolites in Table 1. Upon comparing the inhibition rates, five compounds—quercetin, luteolin, kaempferol, epicatechin gallate, and ellagic acid—exhibited significant LDH inhibitory activity, with stronger inhibition against LDHA than LDHB (Figure 5). To further characterize the inhibitory properties, components exhibiting inhibition rates exceeding 80% were subjected to dose-response evaluation. Quercetin, luteolin, and kaempferol—each active against both isoforms—along with epicatechin gallate, which was active against LDHA, were selected for IC_50_ determination. In Figure 6, the comparison of IC_50_ values revealed that kaempferol was more than 10-fold more potent in inhibiting LDHA (0.70 ± 0.13 µM) than LDHB (8.69 ± 0.85 µM). Similarly, both quercetin and luteolin exhibited a marked preference for inhibiting LDHA over LDHB, with IC_50_ values of 0.27 ± 0.02 µM and 1.19 ± 0.09 µM for LDHA, compared to 0.87 ± 0.07 µM and 2.71 ± 0.39 µM for LDHB, respectively. Although robust activity against LDHA and LDHB was confirmed in this study, the potential for off-target effects in subsequent experiments should be considered due to the known nonspecificity of some polyphenols. Collectively, these findings underscore the potential of *R. crenulata* as a valuable source of LDH inhibitors. Furthermore, these natural scaffolds offer promising starting points for further optimization to enhance potency or isoform specificity.

### 2.6. Molecular Docking of Inhibitory Components to LDHA and LDHB

Molecular docking is a computational approach that predicts the optimal binding mode between a receptor and a ligand by evaluating their geometric, energetic, and chemical complementarity. This technique plays a pivotal role in drug discovery and the investigation of potential therapeutics. Binding energy and key intermolecular interactions serve as the primary indicators for evaluating binding affinity. Lower binding energy values correspond to greater binding stability and stronger molecular interactions. Typically, docking energies ≤ −5.0 kcal/mol suggest substantial binding activity, while values ≤ −7.0 kcal/mol indicate highly strong binding affinity [54]. For intermolecular interactions, given the critical role of hydrogen bonds and hydrophobic interactions in complex stability, their occupancy and surface area were analyzed as key descriptors [55].

To elucidate the interactions of five *R. crenulata* components with LDH isoenzymes, molecular docking simulations were performed against *homo sapiens* LDHA and LDHB crystal structures. The binding energies of all five compounds are summarized in Table 2. Each compound exhibited a binding energy below −5.0 kcal/mol, and 80% of them exhibited values less than −7.0 kcal/mol, demonstrating their robust binding potential. Comparative analysis showed that all five compounds had consistently lower binding energies with LDHA than with LDHB, revealing stronger and more stable interactions with LDHA. This discrepant binding finding aligned well with the results from the enzymatic activity assays.

**Figure 7:**
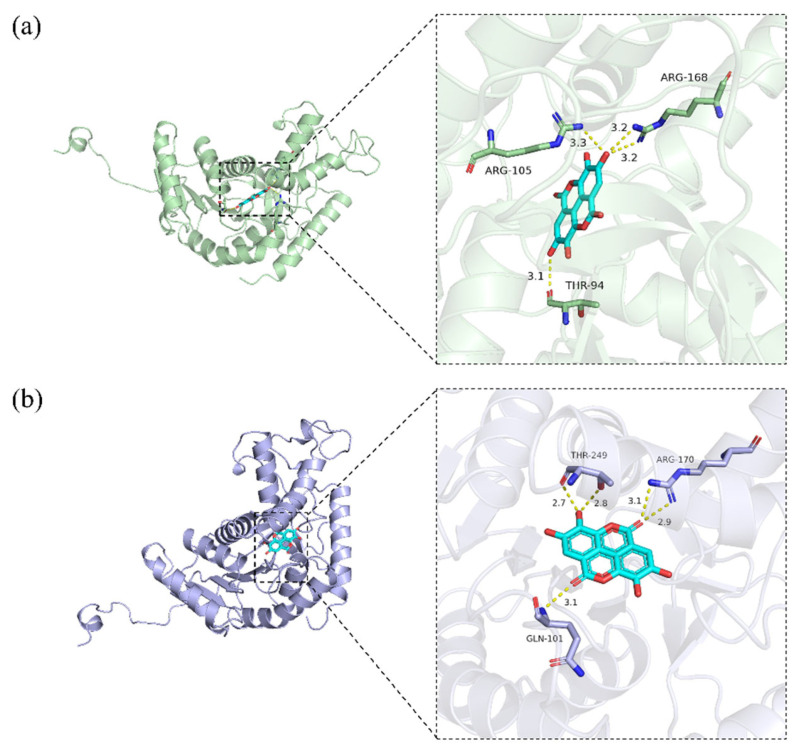
Binding modes of ellagic acid with LDHA (**a**) and LDHB (**b**).

These findings align with prior reports that certain compounds or their structural analogues can act as effective LDH inhibitors. For instance, flavonoids such as quercetin, luteolin, and kaempferol share a common core structure that facilitates multi-modal binding through π–π stacking and hydrogen bonding, resulting in effective LDH inhibition [56]. Similarly, epicatechin gallate features a catechin skeleton, a structure known for its antioxidant and anticancer properties. This skeleton enables the formation of multiple hydrogen-bonding interactions. In addition, the compound’s galloyl moiety may provide additional binding sites that enhance enzyme affinity [57]. Although ellagic acid’s LDH inhibitory activity has been previously reported [58], the molecular docking of its core scaffold to LDH has not been elucidated. Featuring multiple phenolic hydroxyl groups, two lactone groups, and a biphenyl scaffold, it is proposed to bind within the substrate-binding sites of both LDHA and LDHB, as illustrated in Figure 7. Comparative analysis revealed that ellagic acid forms hydrogen bonds with Arg168 in LDHA and the corresponding conserved residue Arg170 in LDHB—both critical for substrate recognition in each subunit. Furthermore, LDHA establishes specific interactions with Thr94 and Arg105, while LDHB forms unique contacts with Gln101 and Thr249. These structural observations indicate that although ellagic acid targets a conserved arginine critical for substrate recognition in both subunits, the distinct microenvironments of the LDHA and LDHB substrate-binding sites likely underlie its differential binding and inhibitory effects. Although ellagic acid does not exhibit high isoenzyme specificity, it provides a valuable structural foundation for the rational design of more selective LDH inhibitors.

### 2.7. Anti-Proliferative Effects of R. crenulata Bioactives on MDA-MB-231 and HepG2 Cells

To evaluate the anti-proliferative efficacy of the five *R. crenulata* components, we employed two human carcinoma cell lines, MDA-MB-231 (triple-negative breast adenocarcinoma) and HepG2 (hepatocellular carcinoma). These models were selected based on the reported divergent roles of LDHB, which exhibits tumor-promoting effects in breast cancer but demonstrates tumor-suppressive activity in liver cancer.

**Figure 8:**
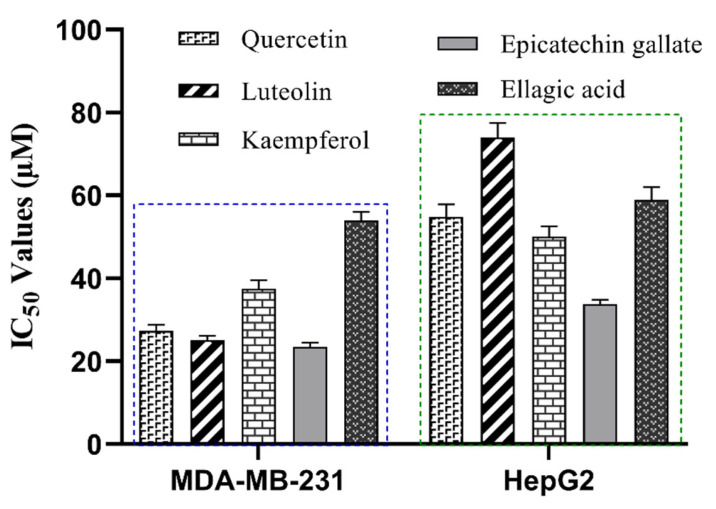
Anti-proliferative activity of five inhibitory compounds against MDA-MB-231 and HepG2 cell lines. The IC_50_ values were consistently lower in MDA-MB-231 cells (blue) than in HepG2 cells (green), demonstrating greater potency against the breast cancer cell line.

As shown in Figure 8, all five compounds demonstrated significantly stronger anti-proliferative effects in MDA-MB-231 cells than in HepG2 cells. The IC_50_ values against MDA-MB-231 for luteolin, quercetin, kaempferol, epicatechin gallate, and ellagic acid were 25.1 ± 1.0 µM, 27.3 ± 1.5 µM, 37.5 ± 2.0 µM, 23.5 ± 1.0 µM, and 54.3 ± 2.5 µM, respectively. The corresponding IC_50_ values against HepG2 were 74.0 ± 3.5 µM, 54.8 ± 3.0 µM, 50.0 ± 2.5 µM, 33.8 ± 1.0 µM, and 59.0 ± 3.0 µM. We propose that this differential efficacy may stem from the distinct LDHB dependencies of the two cell lines. MDA-MB-231 cells are known to be dependent on both LDHA and LDHB for proliferation [59,60]. As our enzyme-level assays confirmed the dual inhibition of both isoforms, the superior anti-proliferative activity in MDA-MB-231 is likely attributable to this concurrent targeting. In contrast, the reduced potency in HepG2 cells is consistent with reports that LDHB expression has no significant impact on their proliferative [21]. Therefore, these cell-based assays functionally validate the anti-proliferative potential of the inhibitors, and the results are consistent with their established role as dual LDHA/LDHB inhibitors.

## 3. Materials and Methods

### 3.1. Reagents and Materials

Referring to the mRNA sequence of *homo sapiens* LDHA (NCBI RefSeq NM_005566.4) and LDHB (NCBI RefSeq NM_001174097.3), the LDHA_pET-28a (+) and LDHB_pET-28a (+) plasmids were synthesized by GenScript Biotech Corporation (Piscataway, NJ, USA). BL21(DE3) competent cells, nickel-nitrilotriacetic acid (Ni-NTA) Sepharose 6FF (His-Tag), kanamycin sulfate, isopropyl β-D-thiogalactoside (IPTG), β-Nicotinamide adenine dinucleotide disodium salt (β-NADH Disodium Salt), and sodium pyruvate were purchased from Sangon Biotech (Shanghai) Co., Ltd. (Shanghai, China). Salidroside, kaempferol, and mycophenolic acid were purchased from Adamas Reagent Co., Ltd. (Shanghai, China). Protocatechuic acid, caffeic acid, (−)-epicatechin, ellagic acid, epicatechin gallate, luteolin, quercetin, tyrosol, and rhodiosin were purchased from Shanghai Standard Technology Co., Ltd. (Shanghai, China). Crenulatin was purchased from Chengdu Push Bio-technology Co., Ltd. (Chengdu, China). Citric acid, gossypol, gallic acid, and azelaic acid were purchased from Shanghai yuanye Bio-Technology Co., Ltd. (Shanghai, China). Astragalin and afzelin were purchased from Shanghai Macklin Biochemical Technology Co., Ltd. (Shanghai, China). Rhodionin was purchased from Shanghai TOP Bio-Pharma Technology Co., Ltd. (Shanghai, China). The MDA-MB-231 and HepG2 cell lines were obtained from Shanghai Institute of Biochemistry and Cell Biology, Chinese Academy of Sciences (Shanghai, China). The Cell Counting Kit-8 (CCK-8) were purchased from Beyotime Biotech Inc. (Shanghai, China). Dulbecco’s Modified Eagle Medium (DMEM), 0.25% trypsin-EDTA, and fetal bovine serum (FBS) were purchased from Thermo Fisher Scientific Inc. (Waltham, MA, USA). The herb *R. crenulata* (Hook. f. et Thoms) H. Ohba was purchased from Yinfeng Chinese Medicine Port (Yulin, China) and authenticated by Professor Lihong Wu.

### 3.2. Instruments and Software

An Agilent 1290 high-performance liquid chromatography system coupled with an Agilent 6545 UHD quadrupole time-of-flight mass spectrometer (HPLC-QTOF MS; Agilent Technologies Co., Santa Clara, CA, USA) was used to analyze the components in *R. crenulata*. Chromatographic separation was performed on a Waters ACQUITY HSS T3 column (2.1 mm × 100 mm, 1.8 μm) with a mobile phase consisting of (A) 0.1% formic acid in deionized water and (B) acetonitrile, delivered at 0.3 mL/min. The gradient elution program was as follows: 0–2 min, 5% B; 2–14 min, 5–25% B; 14–21 min, 25–40% B; 21–28 min, 40–75% B; 28–33 min, 75–95% B; 33–35 min, 95% B. The column temperature was maintained at 40 °C, and the injection volume was 1 µL. The QTOF MS was equipped with a Dual AJS ESI source. And data were acquired in both positive and negative ionization modes. The ESI parameters were optimized as follows: nitrogen was used as the sheath gas at 12 L/min and 325 °C, nebulizer gas at 40 psig, and drying gas at 10 L/min and 325 °C. The capillary voltage, nozzle voltage, and fragment voltage were 4, 0.5, and 0.12 kV, respectively. MS and MS/MS spectra were acquired in the range of *m*/*z* 50–1500. For MS/MS analysis, nitrogen was used as the collision gas with collision energies of 10, 20, and 35 eV. Mass calibration was performed with Agilent ESI-L Low Concentration Tuning Mix (*m*/*z* 100–3200, Santa Clara, CA, USA), achieving a mass resolution of ~25,000 at *m*/*z* 322.

All data were collected and processed using MassHunter LC/MS Data Acquisition B.08.00 (Agilent Technologies Co., Santa Clara, CA, USA) and MassHunter Qualitative Analysis B.07.00 (Agilent Technologies Co., Santa Clara, CA, USA).

### 3.3. Preparation of R. crenulata Extract

The dried roots of *R. crenulata* were ground into powder. And 0.5 g of weighed powder was mixed with 10 mL of methanol in a 15 mL centrifuge tube. The mixture was vortexed vigorously and then allowed to sonicate for 30 min. After centrifugation, the supernatant was carefully collected as the extract and stored at −20 °C for subsequent analysis.

### 3.4. Expression and Purification of Recombinant LDHA and LDHB

The recombinant LDHA-pET-28a (+) and LDHB-pET-28a (+) plasmids were transformed into BL21(DE3) competent cells using the heat shock method. Transformed cells were plated on kanamycin-containing solid medium and incubated overnight at 37 °C. Selected single colonies were inoculated into 5 mL of LB medium with kanamycin and grown overnight at 37 °C with shaking, then expanded to 1 L. When the optical density at 600 nm (OD_600_) reached 0.6–0.8, protein expression was induced by adding IPTG to a final concentration of 0.2 mM. Induction was carried out at 28 °C for 6 h with continuous shaking. Cells were harvested by centrifugation and lysed using a high-pressure homogenizer. The lysate was subjected to Ni-NTA affinity chromatography for initial purification. The eluted protein was subsequently desalted using a desalting column. Further purification was performed using the ÄKTA FPLC system to obtain high-purity recombinant protein. Finally, the purified LDHA and LDHB proteins were aliquoted and stored at −80 °C for future use.

### 3.5. Establishment and Optimization of the LDHA and LDHB Inhibitor Screening Platform

Based on comprehensive literature evidence [41,61,62,63,64] and systematic experimental validation, we established optimized enzymatic assay platforms for LDHA and LDHB inhibitor screening. In the platforms, 60 µL of purified enzyme solution (1.95 nM final concentration) was loaded into 96-well plates, followed by 140 µL of a 1:1 (*v*/*v*) pyruvate/NADH substrate mixture. Reactions were conducted in 0.1 M Tris-HCl buffer (with 10% glycerol, pH 7.4) at 30 °C, and NADH oxidation was kinetically monitored at 340 nm for 20 min. To determine the kinetic parameters, the optimization protocol for LDHA was performed as follows: with NADH fixed at 0.6 mM while varying sodium pyruvate (0.02–2.5 mM) to determine the optimal concentration; and with sodium pyruvate fixed at 1 mM while varying NADH (0.01–0.7 mM) to determine the optimal concentration. Similarly, the optimization protocol for LDHB was performed as follows: with NADH fixed at 0.6 mM while varying sodium pyruvate (0.01–1.05 mM) to determine the optimal concentration; and with sodium pyruvate fixed at 1 mM while varying NADH (0.01–0.7 mM) to determine the optimal concentration. The initial reaction rates were derived by analyzing the progress curves with OriginPro 2023 (OriginLab Co., Northampton, MA, USA). Kinetic parameters (K_m_ and V_max_) were derived by nonlinear regression analysis of the Michaelis-Menten equation using GraphPad Prism 8.0 (GraphPad Software, San Diego, CA, USA).

### 3.6. Evaluation of the LDHA and LDHB Inhibitor Screening Platform

The Z’ factor (Z’ = 1 − [3 × (σₚ + σₙ)]/|μₚ − μₙ|) was used to evaluate the robustness of the screening platform, where σ represents the standard deviation, μ represents the mean value, p denotes enzyme activity, and n denotes the background signal. Gossypol was employed as a reference inhibitor for further validation. The detailed steps were as follows: (1) 59 µL of LDHA/LDHB was added into a 96-well plate, followed by 1 µL of serially diluted gossypol solution (11-point concentration gradient, 0.001–60 µM). (2) The enzyme-inhibitor mixture was pre-incubated for 10 min at room temperature. (3)140 µL of the 1:1 (*v*/*v*) substrate mixture was added (for LDHA: 0.16 mM sodium pyruvate and 0.04 mM NADH; for LDHB: 0.08 mM sodium pyruvate and 0.04 mM NADH). (4) At 30 °C, the enzymatic reaction was monitored at 340 nm for 20 min. The half maximal inhibitory concentration (IC_50_), defined as the inhibitor concentration required to reduce enzyme activity (or cell growth) by 50%, was calculated using nonlinear regression analysis of the dose-response data.

### 3.7. Screening of Inhibitory Components from R. crenulata Against LDHA and LDHB

For the enzyme activity assay, the concentrations of the *R. crenulata* extract were 20 µg/mL, while the 19 isolated components was assayed at 10 µM and 40 µM. DMSO served as the vehicle control, while the assay procedure was identical to that described above for gossypol. The inhibition rate (I%) of *R. crenulata* (or each component) was calculated as I% = (1 − V_i_/V_0_) × 100%, where V_i_ represents the initial enzyme activity rate after treatment with *R. crenulata* (or each component), and V_0_ denotes the initial enzyme activity rate of the control group. Components showing inhibition rates greater than 80% were selected for dose-response analysis.

### 3.8. Molecular Docking Analysis of LDHA and LDHB Inhibitors

The crystal structures of *homo sapiens* LDHA (PDB ID: 1I10) and LDHB (PDB ID: 7DBK) were retrieved from the RCSB Protein Data Bank (https://www.rcsb.org/, accessed on 13 May 2025). Protein preparation, including hydrogen addition and charge assignment, was performed using PyMOL 3.0 (Schrödinger, LLC, New York, NY, USA) and AutoDock Tools 1.5.7 (the Scripps Research Institute, La Jolla, CA, USA). Inhibitory components were obtained from the PubChem database (https://pubchem.ncbi.nlm.nih.gov/, accessed on 13 May 2025) and prepared for docking using AutoDock Tools 1.5.7. Molecular docking simulations were carried out using AutoDock Vina 1.1.2 (the Scripps Research Institute, La Jolla, CA, USA). The docking active site was defined based on literature [65,66,67] to encompass the NADH and substrate-binding pockets. For each inhibitor, 20 independent docking runs were performed, and the optimal pose was selected based on the most favorable binding affinity (lowest score). The protein-ligand interactions were visualized in three dimensions using PyMOL 3.0.

### 3.9. Cell Viability Assays of LDHA and LDHB Inhibitors

The MDA-MB-231 and HepG2 cell lines, representing triple-negative breast cancer and hepatocellular carcinoma, were selected to evaluate the inhibitory effects of LDHA and LDHB inhibitors. Cells in the logarithmic growth phase were seeded into 96-well flat-bottom plates at densities of 5000 cells/well for MDA-MB-231 and 10,000 cells/well for HepG2. After 24 h attachment period, the medium was replaced with 100 µL of fresh medium containing test compounds (8-point concentration gradient, 1.5625–100 µM), or vehicle control (0.5% DMSO). Following 72 h of incubation, 10 µL of CCK-8 reagent was added to each well, and cells were further incubated for 1–4 h. Absorbance was measured at 450 nm with a reference wavelength of 600 nm for background correction. Data from three independent experiments (each with three technical replicates) were normalized to the vehicle control (100% viability) and medium-only wells (0% viability). Dose-response curves were fitted using a four-parameter logistic (4PL) model in GraphPad Prism, from which the IC_50_ values for each compound-cell line combination were derived by nonlinear regression.

## 4. Conclusions

Systematically elucidating the anticancer mechanisms and identifying the active constituents of *R. crenulata* are essential for enhancing its clinical applicability and promoting broader acceptance. In this study, we observed differential inhibition of *R. crenulata* extract against LDH isoenzymes, which prompted further profiling of its inhibitory components. By integrating target-centric enzymology, untargeted metabolomics, structure-guided modeling and functional cellular assays, we established *R. crenulata* as a rich source of LDH inhibitors. The results showed that quercetin, luteolin, kaempferol, epicatechin gallate, and ellagic acid inhibited both LDHA and LDHB, with stronger activity observed against LDHA. We acknowledge that the *in vitro* nature of this work limits insights into bioavailability, metabolism, and *in vivo* toxicity, and that the current preferential inhibition does not equate to clinical selectivity. Despite these limitations, this study provides a comprehensive inhibition profile of *R. crenulata* targeting LDH isoenzymes. The potent *in vitro* activity and structure-activity relationships established here provide a critical foundational basis for future *in vivo* evaluation. Moreover, the preferential inhibition exhibited by five compounds offers a pharmacological starting point for further optimization toward achieving greater isoform specificity for therapeutic applications.

## Figures and Tables

**Table 1:** 36 identified bioactive components in *R. crenulata*.

No.	RT (min)	Precursor Ion		MS/MS (Da)	Formula	Identification	Reference
		Observed Mass (Da)	Error (ppm)				
1	1.349	191.0194 [M−H]^−^	1.7	(−): 129.0188, 111.0086, 87.0084, 85.0292, 67.0187, 57.0346	C_6_H_8_O_7_	Citric acid	standard
2	2.19	169.0145 [M−H]^−^/171.0291 [M+H]^+^	−1.49/−1.77	(−): 125.0243, 79.0191, 51.0244; (+): 153.0181, 127.0389, 125.0229, 109.0285, 107.0126, 81.0335, 53.0388	C_7_H_6_O_5_	Gallic acid	standard
3	4.296	153.0190 [M−H]^−^/155.0341 [M+H]^+^	2.16/−0.75	(−): 108.0212, 109.0294, 110.0325, 91.0189, 81.0345, 53.0398; (+): 137.0227, 93.0335, 111.0440, 81.0333, 65.0387, 53.0383	C_7_H_6_O_4_	Protocatechuic acid	standard
4	4.596	313.0924 [M−H]^−^	1.56	(−): 151.0394, 161.0448, 107.0501	C_14_H_18_O_8_	2-(β-D-glucopyranosyloxy)-1-(4-hydroxyphenyl)ethanone	[46]
5	5.570	461.1660 [M−H]^−^	0.97	(−): 299.1124, 179.0554, 119.0345, 101.0243, 89.0244	C_20_H_30_O_12_	2-(4-hydroxyphenyl)ethyl-6-0-β-D-glucopyranosyl-β-D-glucopyranoside	[47]
6	5.927	345.1199 [M+HCOO]^−/^318.1556 [M+NH_4_]^+^	−2.65/−2.9	(−): 299.1132, 89.0245, 119.0496, 179.0559, 59.0141, 71.0138, 101.0244, 113.0240; (+):69.0335, 85.0285, 121.0649, 145.0496, 187.0751, 205.0856, 229.0858, 247.0961, 265.1070	C_14_H_20_O_7_	Salidroside	standard
7	6.169	293.1252 [M+HCOO]^−^/271.1156 [M+Na]^+^	−4.07/−1.57	(−): 247.1174, 161.0448, 101.0234, 71.0134, 59.0147; (+): 201.0367, 203.0514	C_11_H_20_O_6_	Crenulatin	standard
8	6.468	137.0597 [M−H]^−^	7.99	(−): 119.0501, 106.0420	C_8_H_10_O_2_	Tyrosol	standard
9	7.667	322.1866 [M+NH_4_]^+^	−1.88	(+): 125.0962, 107.0856	C_14_H_24_O_7_	Creoside I or Isomer	[48]
10	8.050	179.0348 [M−H]^−^	1.01	(−): 134.0368, 135.0445, 107.0501	C_9_H_8_O_4_	caffeic acid	standard
11	8.100	322.1866 [M+NH_4_]^+^	−1.88	(+): 125.0962, 107.0856	C_14_H_24_O_7_	Creoside I or Isomer	[48]
12	8.333	577.1347 [M−H]^−^	0.78	(−): 407.0768, 289.0705, 125.0241	C_30_H_26_O_12_	Procyanidin B3	[49]
13	9.073	289.0715 [M−H]^−^/291.0868 [M+H]^+^	0.9/−1.67	(−): 245.0811, 221.0818, 203.0702, 151.0391, 123.0446, 109.0290; (+): 207.0648, 165.0545, 147.0438, 139.0387, 123.0440, 111.0437	C_15_H_14_O_6_	(−)-epicatechin	standard
14	9.615	635.0885 [M−H]^−^	0.77	(−): 483.0785, 423.0574, 313.0560, 169.0139, 125.0242	C_27_H_24_O_18_	trigalloyl-β-D-glucopyranose/isomer	[50]
15	10.239	729.1455 [M−H]^−^	0.83	(−): 577.1325, 451.1040, 407.0774, 289.0718, 169.0143, 125.0248	C_37_H_30_O_16_	catechin-(4α-8)-catechin-3′0-gallate/epicatechin-(4β-8)epicatechingallate	[49]
16	10.489	609.1461 [M−H]^−^	0.01	(−): 446.0854, 283.0242, 151.0031	C_27_H_30_O_16_	kaempferol 3,7-digalactoside/Isomer	[50]
17	10.722	399.1296 [M−H]^−^	0.18	(−): 329.0515, 271.0453, 169.0141, 125.0241	C_18_H_24_O_10_	3-O-galloyl quinic acid butyl ester/Isomer	[51]
18	11.201	787.0993/[M−H]^−^	0.82	(−): 635.0906, 617.0788, 465.0676, 169.0139, 295.0453	C_34_H_28_O_22_	tetragalloyl-β-D-glucopyranose/isomer	[50]
19	11.604	881.1566/[M−H]^−^	0.53	(−): 729.1447, 711.1360, 577.1328, 559.1244, 541.1143, 407.0768, 289.0715, 169.0136	C_44_H_34_O_20_	epicatechingallate-(4β-8)-epicatechingallate	[50]
20	11.962	300.9989/[M−H]^−^	0.3	(−):283.9956, 257.0090, 245.0088, 228.0048, 201.0182, 185.0236, 161.0231, 145.0289, 129.0347, 117.0342	C_14_H_6_O_8_	Ellagic acid	standard
21	11.970	441.0825 [M−H]^−^/443.0979 [M+H]^+^	0.5/−1.42	(−): 331.0459, 289.0708, 271.0605, 245.0817, 169.0140, 125.0243; (+): 291.0857, 273.0755, 207.0646, 165.0548, 153.0179, 139.0390, 123.0442, 111.0437	C_22_H_18_O_10_	epicatechin gallate	standard
22	13.702	447.0931 [M−H]^−^/449.1085 [M+H]^+^	0.41/−1.48	(−): 284.0318, 255.0292, 227.0339; (+): 287.0553, 153.0174, 85.0282	C_21_H_20_O_11_	Astragalin	standard
23	14.084	187.0976 [M−H]^−^	−0.09	(−): 169.0872, 143.1072, 125.0969, 102.9481, 97.0658, 57.0341	C_9_H_16_O_4_	Azelaic acid	standard
24	15.025	881.1566 [M−H]^−^	0.53	(−): 729.1447, 711.1348, 577.0993, 559.1236, 541.1141, 433.0921, 407.0768	C_44_H_34_O_20_	epicatechingallate-(4β-8)-epicatechingallate	[50]
25	15.158	491.2131 [M+HCOO]^−^	0.67	(−): 313.1649, 161.0460, 149.0454, 131.0346, 101.0243	C_21_H_34_O_10_	sachaloside II	[44]
26	15.383	431.0983 [M−H]^−^/433.1136 [M+H]^+^	0.16/−1.57	(−): 284.0322, 285.0390, 255.0289, 227.0339; (+): 287.0550, 147.0649, 129.0546, 111.0434, 85.0282, 71.0492, 57.0333	C_21_H_20_O_10_	Afzelin	standard
27	15.708	609.1459 [M−H]^−^/611.1613 [M+H]^+^	0.34/−1.05	(−): 301.0351; (+): 449.1076, 303.0502, 145.0489, 127.0381, 97.0282, 85.0281	C_27_H_30_O_16_	rhodiosin	standard
28	16.182	447.0933 [M−H]^−^/449.1086 [M+H]^+^	−0.03/−1.7	(−): 301.0346, 255.0289, 229.0505, 166.9975; (+): 303.0503, 169.0124, 121.0276	C_21_H_20_O_11_	Rhodionin	standard
29	17.422	285.0403 [M−H]^−^/287.0555 [M+H]^+^	0.56/−1.7	(−): 199.0391, 133.0294; (+): 269.0429, 241.0494, 213.0539, 153.0179, 135.0438, 117.0328, 89.0389, 67.0179	C_15_H_10_O_6_	luteolin	standard
30	17.439	301.0350 [M−H]^−^/303.0502 [M+H]^+^	1.25/−0.9	(−): 178.9978, 151.0034, 121.0288, 107.0136, 83.0136, 65.0034; (+): 257.0453, 229.0498, 201.0548, 153.0182, 137.0238	C_15_H_10_O_7_	Quercetin	standard
31	17.572	431.0984 [M−H]^−^	−0.07	(−): 285.0394, 257.0457, 151.0039	C_21_H_20_O_10_	kaempferol-7-0-α-L-rhamnoside	[52]
32	17.788	469.2291 [M+HCOO]^−^	−0.12	(−): 423.2232, 291.1811, 161.0430, 101.0243	C_19_H_36_O_10_	rhodiooctanoside	[44]
33	19.828	285.0410 [M−H]^−^/287.0554 [M+H]^+^	−1.88/−1.35	(−): 257.0431, 239.0342, 211.0396, 185.0606, 171.0445, 159.0449, 107.0137, 93.0347, 63.0245; (+): 258.0527, 213.0547, 185.0595, 153.0182, 137.0231, 121.0281, 93.0335, 68.9973	C_15_H_10_O_6_	kaempferol	standard
34	22.733	319.1188 [M−H]^−^/321.1338 [M+H]^+^	−0.28/−1.67	(−): 287.0915, 275.1281, 245.0814, 205.0503, 191.0348, 179.0340, 161.0594, 148.0522, 135.0427; (+): 303.1229, 275.1275, 223.0597, 207.0650, 177.0542, 159.0442	C_17_H_20_O_6_	Mycophenolic acid	standard
35	23.165	479.0986 [M−H]^−^	−0.48	(−): 299.0193, 271.0246, 165.9900	C_25_H_20_O_10_	crenulatin A/crenulatin B/rhodiolin	[51]
36	28.085	295.2278 [M−H]^−^	0.23	(−): 277.2168, 195.1385, 59.0137	C_18_H_32_O_3_	9-hydroxy-10,12-octadecadienoic acid	GNPS

**Table 2:** Binding energies of five inhibitory compounds against LDHA and LDHB.

Component	Binding Energy (kcal/mol)	
	LDHA (1I10)	LDHB (7DBK)
Quercetin	−8.7	−6.8
Luteolin	−8.8	−7.1
Kaempferol	−8.6	−7.2
Epicatechin gallate	−7.7	−7.0
Ellagic acid	−8.5	−6.8

## Data Availability

Data will be made available upon request.

## Abbreviations

The following abbreviations are used in this manuscript:

HPLC-QTOF MS	High-Performance Liquid Chromatography System Coupled with Quadrupole Time-of-Flight Mass Spectrometer
LDH	Lactate dehydrogenase
LDHA	Lactate dehydrogenase A
LDHB	Lactate dehydrogenase B
NAD^+^	Oxidized nicotinamide adenine dinucleotide
NADH	Reduced nicotinamide adenine dinucleotide
BPC	Base Peak Chromatogram
RT	Retention Time
IC_50_	The half maximal inhibitory concentration
IPTG	Isopropyl β-D-thiogalactoside
DMSO	Dimethyl sulfoxide
